# Shedding light on eDNA: neither natural levels of UV radiation nor the presence of a filter feeder affect eDNA-based detection of aquatic organisms

**DOI:** 10.1371/journal.pone.0195529

**Published:** 2018-04-06

**Authors:** Elvira Mächler, Maslin Osathanunkul, Florian Altermatt

**Affiliations:** 1 Eawag: Swiss Federal Institute of Aquatic Science and Technology, Department of Aquatic Ecology, Dübendorf, Switzerland; 2 Institute of Evolutionary Biology and Environmental Studies, University of Zurich, Zürich, Switzerland; 3 Department of Biology, Faculty of Science, Chiang Mai University, Tumbol Suthep Amphur Muang, Chiang Mai, Thailand; 4 Center of Excellence in Bioresources for Agriculture, Industry and Medicine, Chiang Mai University, Tumbol Suthep Amphur Muang, Chiang Mai, Thailand; University of Hyogo, JAPAN

## Abstract

The use of environmental DNA (eDNA) as a species detection tool is attracting attention from both scientific and applied fields, especially for detecting invasive or rare species. In order to use eDNA as an efficient and reliable tool, however, we need to understand its origin and state as well as factors affecting its degradation. Various biotic and abiotic environmental factors have been proposed to affect degradation of eDNA in aquatic environments and thus to influence detection rates of species. Here, we were interested in two of them, namely UV light, which can break down DNA, and the presence of filter feeders, which can remove DNA and DNA-bound particles. A few, mostly laboratory-based studies have found minor effects of UVB on the degradation of eDNA. Ultraviolet A radiation (UVA), however, has been neglected although it also causes DNA lesions and is 10- to 100-fold more prevalent than UVB when reaching the earth’s surface. Filter feeders are common in aquatic ecosystem, but their effects on eDNA has hitherto been ignored. We conducted a full-factorial aquatic mesocosm experiment under near-natural outdoor conditions manipulating UV radiation as well as the presence of *Dreissena polymorpha*, a strong filter feeder capable of filtering cells or organelles containing DNA. Surprisingly, we found that neither UV radiation nor the presence of the filter feeder affected eDNA-based detection rates of macroinvertebrates, even though the experiment took place in summer when UV radiation intensity and filtration activity is high for the chosen experimental site and conditions. These results, in combination with studies from marine or laboratory settings finding no effect of sunlight and its UV components on the detectability of eDNA, suggest that eDNA based species assessments could be relatively robust with respect to our two factors studied.

## Introduction

Organisms constantly shed DNA into the environment, for example through skin or fecal cells, enabling species assessments through non-invasive sampling [1, 2]. In recent years, researchers successfully extracted DNA from environmental samples to detect the presence of organisms in various environments (e.g., [3, 4, 5]). Subsequently, the use of this environmental DNA (eDNA) as a tool for detection has attracted ample attention from both scientific and applied fields, to assess biodiversity [6–10] and even more so to specifically detect invasive or rare species (e.g., [3, 4, 5, 11]). Many of these studies showed that eDNA provides more sensitive estimates of species’ presence than traditional methods (e.g., [5, 12, 13]). In order to establish eDNA as an efficient and robust detection tool for species, however, we need to have a good understanding of the origin, state, and also degradation of eDNA [14, 15] to optimize sampling schemes that lead to more accurate survey results and species detections. This is especially important when eDNA is used as an early warning tool [12, 16], where management actions are implemented depending on the outcome of such tests.

In aquatic systems, Strickler *et al*. [15] give a conceptual model showing how the three main processes of i) production, ii) transport and iii) removal and degradation of eDNA determine the detection of species. Biotic factors can mainly affect the production rate and lead to variation in detection rates, for example due to the specific life stage of organisms during eDNA sampling [17], their activity [18], or densities [19]. A few studies looked at the transportation of eDNA [20, 21], which is hypothesized to be mainly affected by diffusion and discharge [15]. Finally, both biotic and abiotic factors can affect the degradation of eDNA [14]. Among the most commonly proposed and studied abiotic factors degrading eDNA are ultraviolet radiation, temperature, pH, oxygen, salinity, and sediments, while the primary biotic factor proposed is microbial activity [15, 22].

So far, only a few studies experimentally tested the influence of UV radiation on eDNA degradation, focusing on ultraviolet B radiation (UVB). In marine systems, a recent study [23] placed dialysis bags at two depths resulting in different sunlight exposures. The study showed no effect of sunlight on the decay of eDNA. In freshwater systems, Pilliod et al. [24] showed that eDNA more rapidly declined when small (125 mL) containers were placed in the sun compared to the shade. In a second study, Strickler et al. [15] conducted a laboratory experiment where they manipulated UVB radiation intensity of 4 L mesocosms together with temperature and pH levels in a full factorial design. The study showed that temperature has a strong effect, but that UVB and pH also affect degradation rate, and that their respective effects depend on the levels of the other factors. All these studies focused on UVB radiation, which had been proposed to be an important driver of DNA degradation by Strickler et al. [15]. However, in natural sunlight, UVA is 10- to 100-fold more prevalent than UVB when it reaches the earth’s surface [25]. It is well known that UVB and UVA can cause mutations/lesions on the DNA [26], but the specific mechanisms are less clear for UVA, as it is not directly absorbed by the DNA but acts over secondary pathways [27]. UVA radiation also penetrates deeper into the water column than UVB, and might thus have larger effects on eDNA in aquatic systems [28]. Overall, this motivated us to test the effect of different UV radiation types (UVA and UVB) on degradation and subsequent detectability of eDNA in freshwater systems.

Among the biotic factors, microbial activity is one of the few mechanisms hypothesized to be important for degradation of eDNA, and two studies have tested this [22, 29]. However, larger organisms also may affect eDNA degradation. We specifically hypothesized that filter feeders, which are commonly found in aquatic ecosystems, can directly remove eDNA or particles binding eDNA from the water column. For example, mussels are known to filter large amounts of water and removing suspended particles [30]. This effect can be so strong that water bodies can change from a turbid to a clear water state by the presence of certain mussels, such as the widely distributed and often invasive zebra mussel *Dreissena polymorpha* [31], which are therefore also called ecosystem engineers. For *D*. *polymorpha* it has been shown that the size of particles filtered ranges from 0.4 to 40 μm, which partially includes the size of mitochondria or cells [32] that are known to be the main state of eDNA in the environment [33]. Here, we tested the potentially interacting effects of different UV radiation types (UVA and UVB) and filter feeders on detectability of eDNA under near-natural conditions.

## Material & methods

In a full factorial design, we tested the effect of three different UV-light levels and the presence/absence of a filter feeder on eDNA-based detectability of macroinvertebrates in small standing water bodies, comparable to ponds. To do so, we enriched freshwater mesocosms with the DNA of three common macroinvertebrates (*Gammarus pulex*, *Asellus aquaticus* and *Potamopyrgus antipodarum*), and placed them outside in a natural sunlight regime. We applied different cover materials to manipulate the type of UV radiation reaching the water and manipulated presence/absence of the filter feeding mussel *Dreissena polymorpha* to test their effects on the detectability of macroinvertebrate eDNA. We tested the detectability of the three macroinvertebrates’ eDNA over a time span of six days using standard PCR and species-specific primer pairs.

### Ethics statement

All experiments in this study followed the current laws of Switzerland. We did not need a permit for collecting the four different invertebrate species used in our experiment, as these invertebrate species are not protected by the law nor are they listed as endangered. Furthermore, the species were all present and collected in the catchment where the experiment took place.

### Primer design and specificity test of primers on tissue DNA

We designed species-specific primers for the freshwater amphipod *Gammarus pulex* and the freshwater isopod *Asellus aquaticus*, and used already published primers for the freshwater snail *Potamopyrgus antipodarum* [16], but without the use of the MGB probe (Table 1). For both *G*. *pulex* and *A*. *aquaticus*, we used own generated sequences (GenBank accession numbers are listed in S1 Table) to design the primers. We then used Sequencher® (version 4.9; Gene Codes, Ann Arbor, Michigan, USA) to observe intraspecific conserved regions of the targeted COI gene and compared it to sequences from related species (see S1 and S2 Tables for GenBank accession numbers). We designed the primers with Primer3web (version 4.0.0, [34]) software by using the default settings and improved the suggested primers to maximize base pair differences to closely related species [35]. We checked for the formation of secondary structures of the primers with the free edition of Bacon Designer (PREMIERE Biosoft, California, USA) and chose primers that showed reduced affinity for secondary structures. We optimized the PCR protocol for each primer set with temperature and MgCl_2_ gradients by using tissue extracted DNA (tDNA) of the specific species. We aimed to test all primers in a PCR against tDNA of species belonging to the same genus as the targeted species are known to be present in Switzerland, which was especially relevant for a number of common amphipod species [36]. *Asellus* and *Potamopyrgus* have no other species present locally in the genus other than the target species, thus we extended the test to further-related species (Table 2).

**Table 1:** Used primer pairs to detect species-specific eDNA.

**Target species**	**Primer name**	**Sequence (5’-3’)**	**Amplicon size (bp)**^a^	**Publication**
*Asellus aquaticus*	Aaq-L3	GGCAATGACCAGATTTACAATGTAA	147	Herein
	Aaq-R3	ATTTATACGAGGAAATGCTATATCTGG		Herein
*Gammarus pulex*	Gpu-L9	CTCTAACCCTTCTACTTATAAGTAGTA	179	Herein
	Gpu-R11	GTAGAGATAAAATTAATAGCGCCG		Herein
*Potamopyrgus antipodarum*	NZMSF	TGTTTCAAGTGTGCTGGTTTAYA	89	Goldberg *et al*. 2013
	NZMSR	CAAATGGRGCTAGTTGGAATTCTTT		Goldberg *et al*. 2013

^a ^Amplicon size is including the primer pair length

**Table 2:** List of species that were used to test the specificity of primers.

**Primer target species**	**Closely related species**	**In catchment present**	**Successful amplification**
***Asellus aquaticus***	*Jaera istri*	No	no
	*Proasellus coxalis*	No	yes
***Gammarus pulex***	*Gammarus fossarum*	No	no
	*Gammarus roeseli*	No	no
	*Gammarus lacustris*	No	no
	*Echinogammarus stammeri*	No	no
	*Dikerogammarus villosus*	Yes	no
	*Crangonyx pseudogracilis*	No	yes
***Potamopyrgus antipodarum***	*Lithoglyphus narticoides*	No	no
	*Dreissena polymorpha*	Yes	no

### Target organisms

Species were collected from the field on May 26 and 30, 2016 (Chriesbach, Zurich, Switzerland, latitude 47.40454°N, longitude 8.60898°E). Per species we had five small containers that were filled with tap water (6.5 L). Individuals were then added to the containers to enrich the water with their eDNA (*Gammarus pulex*: 68 individuals per container; *Asellus aquaticus*: 26 individuals per container; *Potamopyrgus antipodarum*: 64 individuals per container). During this incubation period, we fed *G*. *pulex* and *A*. *aquaticus* ad libitum with maple leaves *(Acer* sp.*)* previously incubated in water for 11 days, while *P*. *antipodarum* was fed ad libitum with *Spirulina* sp. (1 teaspoon of powder dissolved in 50 mL of water, of which about 10 mL were added per container and day). We kept the animals for at least 10 days in the containers, and we did not observe any mortality. Water losses due to evaporation were replaced daily. We then filtered the water of each container with a mesh size of 500 μm in order to remove individuals and leaf litter. We mixed the five containers per species together and took an eDNA sample (as described below, see S3 Table for volume and concentrations).

UV radiation may not only affect the degradation of macroinvertebrate eDNA, but also the release of eDNA due to its stressful impact on aquatic organisms [37, 38]. As we only were interested in the former and wanted to exclude different eDNA shedding rates depending on the treatment, we added water which was naturally enriched with the target organisms’ eDNA (see previous paragraph), rather than adding the organisms themselves to the experiment. Therefore, we distributed 1 L of each species’ eDNA-enriched water to each mesocosm at the onset of the experiment.

### Treatments

**Fig 1:**
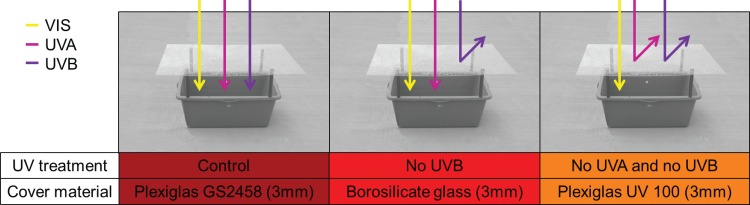
Overview of the three different UV treatments and the specific cover material used. In the control treatment, visible light (VIS) and all wavelengths of UV radiation can penetrate the cover. In the ‘No UVB’ treatment only VIS and UVA radiation can penetrate the cover, while UVB radiation will be reflected. Similar in the ‘No UVA and no UVB’ treatment: UVA and UVB radiation will be reflected and only VIS can reach the water in the mesocosms.

We used different cover materials to manipulate the UV content reaching the water and thus the eDNA in the mesocosms (Fig 1). For the control treatment we used Plexiglas® GS2458 (3mm, Evonik Performance Materials GmbH, Darmstadt, Germany), allowing full penetration of all UV levels. For the exclusion of UVB radiation we used borosilicate glass (3mm, Glas Trösch AG INTERIEUR, Volketswil, Switzerland). For the exclusion of all UV radiation we used Plexiglas® UV 100 (3mm, Evonik Performance Materials GmbH, Darmstadt, Germany). The covers substantially jutted out on all sides over the edge of the mesocosms, such that the sun light could only reach the surface of the water by passing through the respective covers. To half of the mesocosms, we added the mussel *Dreissena polymorpha*, which is known to be a heavy filter feeder [31]. *Dreissena polymorpha* individuals were collected on June 1, 2016 (Greifensee, Zürich, Switzerland, latitude 47.33996°N, longitude 8.67941°E), kept in the laboratory until the distribution of individuals to mesocosms, and fed ad libitum with cultures of the green alga *Scenedesmus* sp. We distributed 15 individuals of *D*. *polymorpha* with varying body size (mean length 8.8 mm ± 2.7 mm standard deviation) randomly to each of the mesocosms belonging to the filter feeder treatment.

In a full-factorial design we tested the effect of UV (three levels) and the presence of a filter feeder (two levels) on eDNA detection rates. Each treatment combination was replicated five times (equals a total of 30 mesocosms) and the treatments were randomly assigned to the mesocosms. During the experiment, we measured UV radiation of the natural sunlight daily with a JAZ-EL200 spectrometer (Ocean Optics, Florida, USA) at 9 a.m., 12 p.m., and 3 p.m., and collected eDNA samples on a daily basis.

### Mesocosms

We used 90 L plastic tanks for our mesocosms and placed them on the rooftop of a research facility of Eawag near Zurich, Switzerland (latitude 47.40521°N, longitude 8.60951°E, 432 m above sea level). We cleaned the mesocosms with 2.5% sodium hypochlorite (i.e. bleach) and thoroughly rinsed them with tap water before use. Afterwards, we filled them with 48 L of tap water which resulted in a water column depth of 0.21 m and a surface area of 0.25 m^2^. We added an aeration system for a constant mixing of the water column (aquarium air stones, Tetra Tec Instruments GmbH, Baden-Württemberg, Germany). To confirm that the material was clean and that there was no background eDNA of our specific target species present, we collected one eDNA sample per mesocosm (t = 0) on May 25, 2016. We added HOBO pendant temperature loggers (Onset, Bourne, Massachusetts, USA) on May 26 and collected temperature data at an hourly interval.

### Environmental DNA sampling, extraction and PCR

We took an eDNA sample from each mesocosm immediately after the addition of the water enriched with the target organisms’ eDNA. Thereafter, we sampled eDNA at a 24 h interval for six days. We wore nitrile disposable gloves while sampling and exchanged them after each mesocosm was sampled to avoid any cross-contamination. Each eDNA sample consisted of 250 mL water per mesocosm, filtered on a single GF/F filter (0.7 μm Whatman International Ltd., Maidstone, UK). We directly sampled water with a 50 mL disposable syringe from the individual mesocosms and filtered on site. After the water filtration, we pushed 50 mL of air through the filter to get rid of excess water on the filter. Then we opened the filter housing and transferred the filter with tweezers to a 1.5 mL tube. The tube was stored in the dark and on ice until all mesocosms were sampled (max. 1 h). Tweezers were cleaned with 2.5% sodium hypochlorite, wiped with 70% Ethanol and dried between the different samples. Thereafter, all tubes were frozen at –20°C until further processing.

We used the Qiagen DNeasy Blood & Tissue kit (Qiagen, Hilden, Germany) for the extractions. Details to the extraction and the performed PCR can be found in the supporting information (S1 Appendix). We performed five PCR replicates per sample and for each PCR plate we included a negative (PCR water) and positive (tDNA) control. Amplification success was tested with the QIAxcel Advances Systems by using a High Resolution Cartridge (Qiagen, Hilden, Germany), the OM500 method and the Default DNA v2.0 analysis. We used the QX 15 b–1 kb alignment marker and the 50–800 bp reference marker (5 ng/μL). A PCR was assigned as positive if there was a clear peak adjacent to the expected amplicon size.

### Sequencing

Due to cross-amplification we randomly selected 10% of all positive eDNA reactions with the right amplicon size per species (for *A*. *aquaticus* 17, *G*. *pulex* 25) and sequenced the PCR product. Further, we sequenced two negative controls (one *for G*. *pulex* and one for *A*. *aquaticus*) that showed a peak in the electropherogram. 5 μL of each PCR product was cleaned with 0.5 μL EXO I and 1 μL rSAP, heated up to 37°C for 15 min followed by 15 min at 80°C. Each PCR product was sequenced in both directions with the Big Dye Terminator (version 3.1) system on 3730xl DNA Analyzer following the provided protocols (Applied Biosystems, Foster City, California USA). A sequence was counted as confirmed if the generated sequence length matched the expected sequence length and the generated sequence matched the anticipated species (≥ 98% maximum identity and ≥ 98% query coverage) when testing against the NCBI database by using default BLAST settings [39].

### Laboratory conditions and negative controls

We took specific and extensive laboratory precautions developed and recommended for the work with eDNA, and described in detail in [11]. In deviation of [11], we here filtered eDNA samples directly in the field. To ensure that this did not cause any contamination, we also included a negative equipment control (EQC) at each sampling event. The EQC consisted of Milli-Q® water that was treated with UV-C light before. The EQC was filtered before the mesocosms, so that can be used as a control to check if our equipment was clean, which is important because syringes and filter housings were reused during the project. Further, we included a negative extraction control (EXC) which consisted of a GF/F filter that was UV radiated prior to its use and then extracted along with eDNA samples. We tested all EQC and EXC samples in five replicates of PCR per primer pair alongside with the eDNA samples.

### Statistics

We used R version 3.3.2 [40] and the R-package lme4 version 1.1–13 [41]. We analyzed the effect of the different UV levels and the presence of a heavy filter feeder on eDNA detection rates using a linear mixed effect model with a binomial error distribution. We used the proportion of positive detections across all five PCR replicates of a specific primer pair as the response variable. In the model, we used the UV and the filter feeder treatments as fixed effects. We treated the day of the experiment and the mesocosm identity as random effects (day | mesocosm) in a random slope and intercept model. We increased the number of function evaluations to 100,000 due to model convergence issues. We compared the models with and without interaction of the fixed effects (UV and the filter feeder treatment) with a mixed model ANOVA analysis and selected the best model based on the AIC criterion. The models were tested for each species separately.

## Results

### Primer specificity

Our two newly designed primers successfully amplified the DNA of the respective targeted species *Gammarus pulex* and *Asellus aquaticus*. While generally specific, both primers also amplify DNA from tissue samples of one closely related non-targeted species each: the Gpu primer pair amplified the tDNA of *C*. *pseudogracilis* and the Aaq primer pair amplified tDNA of *P*. *coxalis* (Table 2). However, both non-target species are not expected to occur in the catchment of our study area, can be excluded as being present in the tap water used in our study, and were not added as individuals to our mesocosms. Thus, an erroneous mis-amplification in the experimental setting is highly unlikely. While any such mis-amplifications would be conservative with our treatment and conclusions, we sequenced a subset of the experimental eDNA based bands (see below) to demonstrate that our eDNA based samples were based on our targeted species. The primers for the *P*. *antipodarum* did not amplify any other species that the primer pair was tested against.

### eDNA detectability

**Fig 2:**
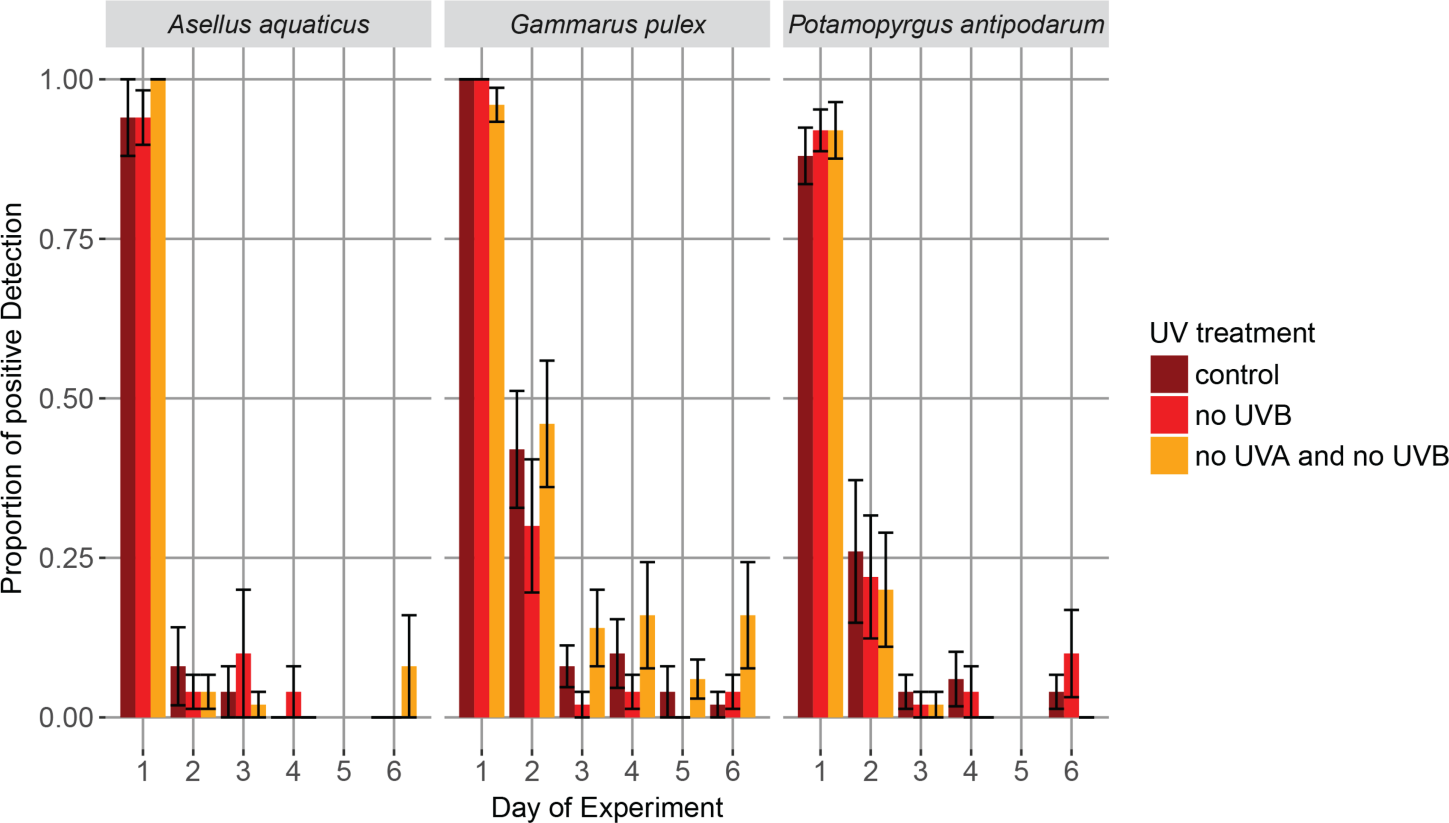
Mean proportion of successful eDNA detection per UV treatment based on positive amplifications across five PCR replicates per mesocosm. Error bars represent standard errors across all ten mesocosm replicates of the corresponding UV treatment. We did not process eDNA samples for day five for *A*. *aquaticus* and *P*. *antipodarum* due to logistic reasons.

**Fig 3:**
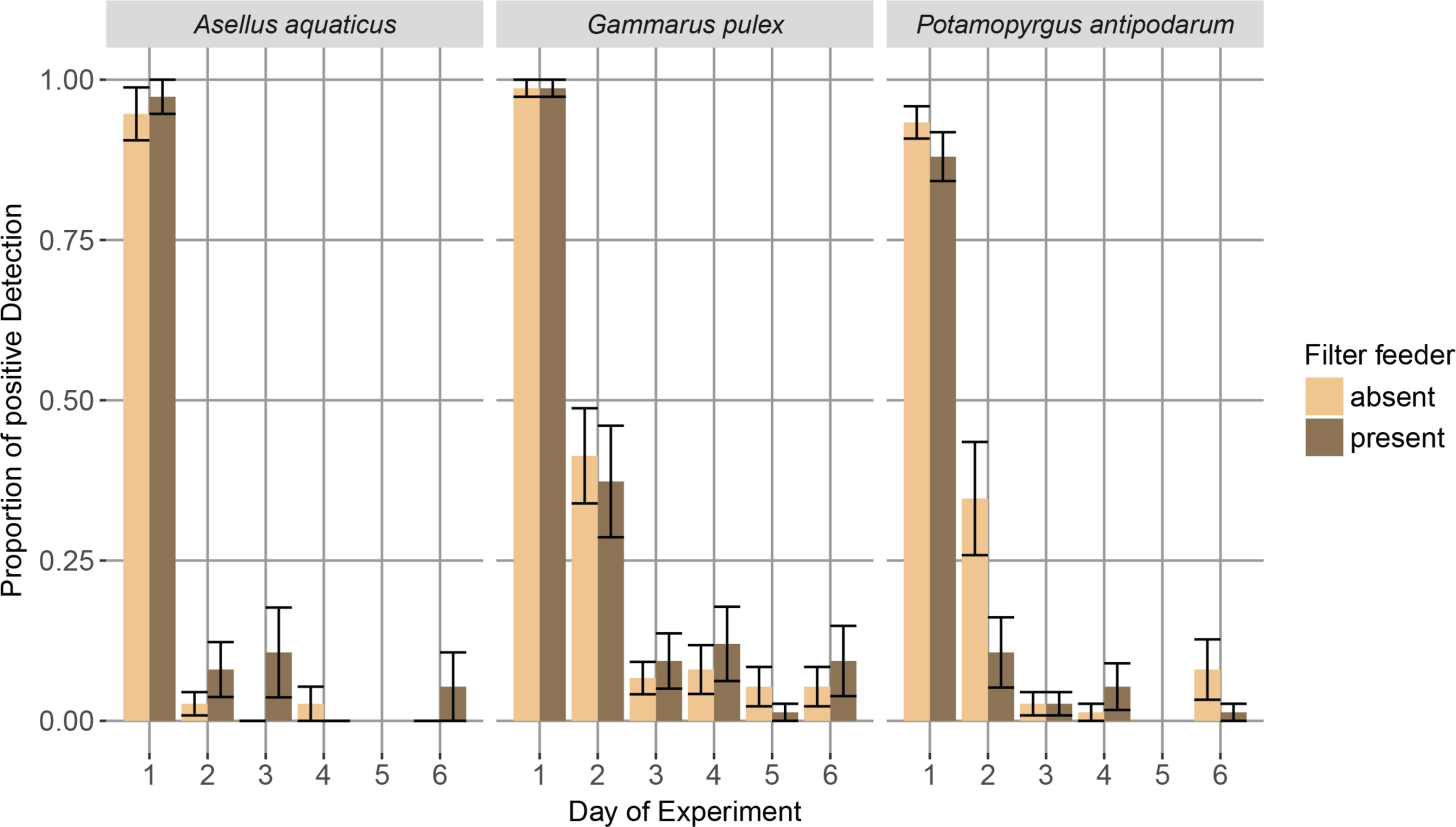
Mean proportion of successful eDNA detection per filter feeder treatment based on positive amplifications across five PCR replicates per mesocosm. Error bars represent standard errors across all 15 mesocosm replicates of the corresponding filter feeder treatment. We did not process eDNA samples for day five for *A*. *aquaticus* and *P*. *antipodarum* due to logistic reasons.

On day one of the experiment we found a very high detection probability of all three targeted macroinvertebrate species in the 250 mL water sample and subsequent eDNA based assessment (mean of 96% ± 2.4% for *A*. *aquaticus*, 98.7% ± 0.9% for *G*. *pulex* and 90.6% ± 2.3% for *P*. *antipodarum* over all treatments, data available in S4 Table). Thereafter, we found that the proportion of positive eDNA detection declined relatively rapidly for all three targeted macroinvertebrate species within the first two days, but for all three species we still found low detection at day six of the experiment, indicating that some eDNA persisted throughout this time period (Fig 2). Contrary to our expectation, however, detection rates were not affected by the UV radiation treatment nor the filter-feeding treatment (or their interaction): we did not find any significant influence on the proportion of eDNA-based detection across the three different UV levels of the UV radiation treatment, and there was no significant difference in eDNA detection between the replicates with or without filter feeders (Tables 3 and 4, Fig 3). All EQC and EXC were blank and did not amplify with any of the primer pairs. Further, all preliminary eDNA tests of mesocosms (t = 0) were blank, showing that the water used for filling the mesocosms was free from detectable amounts of eDNA from the specific targeted species. All but two negative PCR controls were blank. We sequenced the two negative controls that amplified, one belonging to a *Gammarus pulex* PCR reaction and one belonging to *Asellus aquaticus* PCR reaction. The sequenced negative control for *Gammarus pulex* did not meet the confirmation criteria stated above. The sequencing quality was very low (0.9%), indicating that no or only a poor-quality DNA template was present in the sequencing reaction. The sequence for the negative PCR control for *Asellus aquaticus* met the confirmation criteria above and likely happened due to cross-contamination since we were pipetting the positive control right next to the well of the negative PCR control. We included the PCR replicates generated with the same master mix, as no other PCR reaction besides the negative and positive control amplified. Thus we treated both amplifications of the negative PCR controls as negative results. The sequencing of the eDNA samples was successful: all amplicons met the stated confirmation criteria and matched the respective targeted species, and were counted as positive eDNA detections.

**Table 3:** GLMM results on the fixed effects of the UV and filter feeder treatments on the detection of each species.

	**Coefficient**	**Standard error**	**Z value**	**p value**
***Asellus aquaticus***				
Intercept (UV: Control)	-0.7779	0.8429	-0.92	0.36
UV: No UVB	0.3861	0.9113	0.42	0.67
UV: No UVA and no UVB	-0.0991	0.9252	-0.11	0.92
Filter feeder: Present	0.6748	0.7591	0.89	0.37
***Gammarus pulex***				
Intercept (UV: Control)	-0.9799	0.7287	-1.35	0.18
UV: No UVB	-0.2661	0.6355	-0.42	0.68
UV: No UVA and no UVB	0.5955	0.6065	0.98	0.33
Filter feeder: Present	-0.0669	0.5154	-0.13	0.90
***Potamopyrgus antipodarum***				
Intercept (UV: Control)	0.2271	0.8808	0.26	0.80
UV: No UVB	-0.3026	0.7214	-0.42	0.68
UV: No UVA and No UVB	-0.4077	0.7432	-0.55	0.58
Filter feeder: Present	-0.4594	0.5868	-0.78	0.43

**Table 4:** GLMM results of the random effects (mesocosm identity and day of experiment) on the detection of each species.

	**Variance**	**Standard deviation**
*Asellus aquaticus*		
Intercept (mesocosm)	84.98	9.218
Day	32.79	5.726
*Gammarus pulex*		
Intercept (mesocosm)	25.314	5.031
Day	4.484	2.118
*Potamopyrgus antipodarum*		
Intercept (mesocosm)	30.745	5.545
Day	9.905	3.147

The water temperature in the mesocosms was comparable between the three types of covers (i.e., the three UV treatments): mean (± standard error) temperature for the control treatment was 18.9°C ± 0.1°C over the course of the experiment, 18.9°C ± 0.1°C for the ‘No UVB’ treatment and 18.8°C ± 0.1°C for the ‘No UVA and no UVB’ treatment. The maximum temperature observed in a replicate was 31.1°C for the control, 32.0°C for the ‘No UVB’ treatment and 31.1°C for the ‘No UVA and no UVB’ treatment (data available in S5 Table). These maximum temperatures were all measured on day 2 of the experiment. In the mesocosms with the filtration treatment, all but very few *Dreissena* individuals were alive throughout the whole experiment.

## Discussion

We studied if and how natural levels of UV radiation and the presence of filter feeders, common abiotic and biotic factors in aquatic ecosystems, affect detection rates of macroinvertebrate eDNA. Surprisingly, natural levels of UV radiation had no effect on the detection probability of species-specific eDNA, even though the experiment took place in summer when UV radiation intensity is the highest for our experimental site [42]. Additionally, the presence of a heavy filter feeder did not show any effect on the detection of eDNA. Overall, the detectability of eDNA decreased quickly and over timescales comparable to other studies (e.g., [9, 19, 22]) and our results indicate a relatively high robustness of eDNA-based estimates with respect to two major factors hypothesized to affect eDNA detection (UV radiation and filter feeders). Consequently, other factors, such as microbial activity or pH [15, 43], may be the main drivers of eDNA degradation and eDNA detectability over time. Our findings suggest that variation in UV radiation may be negligible for the application of eDNA as a detection method, at least for sampling sites at low altitudes in the temperate zone. Importantly, this may not necessarily be the case for alpine or tropical regions, where UV intensity is generally higher (e.g., [42, 44]). Our results are in accordance with a recent study that tested the difference of sunlight on the degradation of fish eDNA in marine systems [23], where the effect of UV radiation is supposed to be even higher, but also did not affect detectability.

There was no difference in the detection of eDNA between the two treatment levels that either excluded UVB radiation only or excluded both UVA and UVB radiation, which seems to contradict the current understanding of UVB’s role in the degradation of eDNA. Although Strickler *et al*. [15] found that degradation of eDNA was positively associated with UVB intensity, the data showed a non-linear effect and did not include effects of UVA. To our knowledge, our experiment is the first that investigated individual and combined effects of the two UV components (UVA and UVB) on the detectability of eDNA. UVB radiation usually penetrates only a few decimeters into the water column whereas UVA can penetrate deeper, but the penetration depth is dependent on optical properties of the water, such as the dissolved organic carbon (DOC) concentration [28]. Since we used tap water, which had a very low DOC level, and the depth of our water column was only about 0.21 m, there should not be a difference in the penetration depth of the two UV components in our experimental setting.

While the results of our UV treatments are in accordance with some recent studies [23], they contradict the finding of Pilliod *et al*. [24], who detected a faster decline in the ‘sun’ compared to the ‘shade’ treatment (after 4 days, they detected eDNA in 40% of the sun treatment samples compared to 80% in the shade treatment samples). However Pilliod *et al*. [24] did not control for temperature during the experiment. The temperature in the ‘sun’ versus the ‘shade’ treatment may have been different, and temperature has been shown to be an important confounding factor by other studies [15, 29, 45]. In our study, the temperature did not differ between the different mesocosms irrespective of the cover material, and thus we can exclude an effect due to temperature differences. The general rapid decline in eDNA detectability at the beginning of the experiment is consistent with other studies (e.g., [4, 19, 46]), but may also been driven by the relative high ambient temperature during the first two experimental days. We cannot exclude that at lower temperatures, which allow a slower degradation of eDNA [15, 29, 45], a difference between the different UV treatments could have occurred. Thus, it would be interesting to repeat our study in alpine regions, where temperatures are generally lower but UV radiation intensity is higher. While quantitative PCR (qPCR) has been shown to be more sensitive [35], we have used a standard PCR procedure, because the concentrations of our eDNA samples were below the limits of detection (LOD) for the qPCR assay and thus enabled a reliable quantification of DNA. We do not, however, think that our choice of DNA amplification method has biased our results.

The presence of the heavy filter feeder *Dreissena polymorpha* also had no effect on the detectability of the eDNA, although studies have shown that *D*. *polymorpha* can filter up to 0.1 L per hour and individual [31], and thus a single individual could have filtered one-third of the volume of our mesocosm in less than a day. The absence of an effect could be due to three reasons: First, the density of the filter feeder may not have been sufficiently high to have an effect. We used a density of 41 *D*. *polymorpha* individuals m^–2^, which is much lower than naturally observed densities of up to 32,000 individuals m^–2^ [47]. However, these maximal densities may not be the norm. Second, the individuals might have had reduced filtration rates due to the relatively high temperature [48]. The optimal temperature range for adult individuals of *D*. *polymorpha* lies between 20–25°C, which is within the range of our average temperatures across the experiment, but lower than the experimentally observed maximum temperature peaks of up to 32.0°C [49]. Third, *D*. *polymorpha* could also have an indirect positive effect on the stability of eDNA by filtering out bacteria that otherwise would have degraded eDNA. Further, differences between bivalve species in particle size retention [50] could lead to varying impacts. Disentangling such possible pleiotropic effects of filter feeders on eDNA is a hitherto understudied aspect and cannot be disentangle by our approach, as we only looked at the net effect of the presence of *D*. *polymorpha*.

## Conclusion

Our study suggests that neither UV components of natural sunlight nor a common filter feeder interfere with the application of eDNA as a detection tool under realistic semi-natural conditions. Our UV results from an outdoor freshwater mesocosm experiment are in concordance with experiments conducted either in marine systems or studying UV effects in small microcosms [15, 23], which all did not find an effect of UV radiation on eDNA detectability. Importantly, however, we only studied one filter feeder species at a relatively low density, and acknowledge that effects of filter feeders at high densities are possible, either by directly filtering out eDNA or by indirectly affecting density of eDNA-degrading bacteria.

## Supporting information

S1 Appendix Detailed information on the extraction of eDNA samples. (DOCX)

S1 Table GenBank accession numbers for own generated sequences used for primer design. (XLSX)

S2 Table GenBank accession numbers for previously published sequences used for primer design. (XLSX)

S3 Table Filtered volume and concentration for each species-specific container. (XLSX)

S4 Table PCR data for each time point, species and PCR replicate. (XLSX)

S5 Table Temperature data of each mesocosm. (XLSX)
