# Supplementary material for: Shedding light on eDNA: neither natural levels of UV radiation nor the presence of a filter feeder affect eDNA-based detection of aquatic organisms
Source: PLoS One. 2018 Apr 6;13(4):e0195529. doi: 10.1371/journal.pone.0195529 (PMC5889167; doi:10.1371/journal.pone.0195529)
Supplement: S1 Appendix — (DOCX) [file pone.0195529.s001.docx]

**S1 Appendix: Detailed information on extraction of eDNA samples**

We followed the manufacturer’s protocol for animal tissues (spin-column protocol) with the following changes: for all samples we eluted the DNA twice with 50 µL AE buffer, in a total volume of 100 µL. Additionally, for eDNA samples we doubled the volume of ATL buffer (360 µL), Proteinase K (40 µL), AL buffer (400 µL) and Ethanol (400 µL). In order to load the total volume on a spin column, we had to repeat the subsequent centrifugation step (point 4 in the provided protocol) twice. PCR reaction of eDNA samples were set up the following: 1x Probes Master (12.5 µL, Roche Diagnostics, Rotkreuz, Switzerland), 0.5 nM of each primer (1.25 µL), 1 µg/µL BSA (2.5 µL, GeneON, Ludwigshafen am Rhein, Germany), 3 µL DNA template, in a total reaction volume of 25 µL. The PCR regime consisted of 95 °C for six minutes, followed by 50 cycles of denaturation at 95 °C for 30 seconds, annealing at 60 °C (*G. pulex* and *P. antipodarum*) respectively 58 °C for *A. aquaticus* for 30 seconds and elongation at 72 °C for 45 seconds, ending the PCR with a final hold of 72 °C for 7 minutes.
